# Strengths and Limitations of Salicylic Acid Reporters

**DOI:** 10.3390/ijms262110610

**Published:** 2025-10-31

**Authors:** Viktor V. Morozov, Ilia V. Yampolsky, Bulat K. Iskakov, Anastasia V. Balakireva

**Affiliations:** 1 Shemyakin–Ovchinnikov Institute of Bioorganic Chemistry, Russian Academy of Sciences, 117997 Moscow, Russia; viktor@cloning.tech (V.V.M.); ivyamp@gmail.com (I.V.Y.); 2 Institute of Translational Medicine, Pirogov Russian National Research Medical University, Ostrovityanova 1, 117997 Moscow, Russia; 3 Aitkhozhin Institute of Molecular Biology and Biochemistry, Science Committee Ministry of Science and Higher Education, 050012 Almaty, Kazakhstan; bulat.iskakov@mail.ru

**Keywords:** salicylic acid, phytohormone, transcriptional reporters, microbial sensor, fluorescent chemical probe, signaling

## Abstract

Salicylic acid (SA) is a key phytohormone that coordinates plant innate immunity and systemic acquired resistance. Because SA levels and signaling are highly dynamic in space and time, a suite of SA-focused tools, including SA-specific microbial biosensors and SA-responsive transcriptional and chemical reporters, has been developed to study them. This review compares three classes of tools in terms of sensitivity, specificity, temporal resolution, invasiveness, quantifiability, and suitability across species. We describe developing genetically encoded sensors that can directly sense salicylic acid and report it, for example, via a fluorescence resonance energy transfer signal or another real-time output. We offer recommendations on method selection by research goal and plant species, as well as combined protocols (long-term autoluminescence plus local probes/biosensors) for cross-validation. Future work should prioritize substrate-free, quantitative SA reporters deployable in crops and the field; coupled with CRISPR-based editing and screening, these tools would enable reporter-guided discovery of immunity genes and rapid engineering of durable disease resistance.

## 1. Introduction

Salicylic acid (SA) is a central phytohormone orchestrating plant immune responses and stress signaling. SA is a widely distributed phenolic compound derived from the shikimate/phenylpropanoid pathway, and it plays diverse regulatory roles in plant physiology beyond immunity [1]. In addition to its biotic defense functions, SA has been increasingly recognized as a mitigator of abiotic stresses (drought, salinity, temperature extremes, heavy metal toxicity) by modulating major metabolic processes for stress tolerance [2]. For instance, under heavy metal stress, SA can limit metal uptake, boost antioxidant defenses, and protect membrane integrity, thereby alleviating metal-induced damage [3].

SA’s role in plant stress responses is ancient, dating back to early land plant evolution. Evolutionary analyses suggest that the SA signaling pathway evolved in pieces—for example, SA receptors (NPR proteins) appear only in land plants, whereas the transcriptional activators they cooperate with (TGA factors) exist even in algae, and a full isochorismate-based SA biosynthetic pathway was assembled with the emergence of terrestrial plants [4]. Notably, SA is involved not only in plant–pathogen defense but also in beneficial plant–microbe symbioses; SA levels are actively modulated during interactions with endophytes, mycorrhizal fungi, and rhizobia to balance growth and defense [5].

In particular, SA accumulation triggers local defenses and systemic acquired resistance (SAR) against pathogens. Because SA levels and signaling are highly dynamic in time and space, mapping SA activity in planta is crucial for understanding how plant immunity is coordinated. In practical terms, reporter lines (e.g., PR1::LUC/YFP) visualize where SAR is primed and how it spreads: reporter activity first appears near the inoculation site and then expands to distal leaves over ~1–2 days, consistent with systemic signaling dynamics [1]. Complementarily, microbial biosensors quantify early SA availability at infection fronts, while chemical probes resolve cell-scale SA hotspots, linking SAR priming to SA distribution at multiple scales [2,6,7,8,9]. Emerging evidence shows that SA signaling is remarkably complex, involving multiple receptors and the formation of dynamic signaling complexes in cells. This complexity highlights the need for in vivo reporters to capture the spatial and temporal nuances of SA action [10]. For example, Betsuyaku et al. visualized SA’s spatial dynamics via time-lapse imaging: around an infection site, SA forms a steep concentration gradient that activates defense genes (like PR1) only in cells immediately adjacent to the hypersensitive response (HR) lesion, whereas jasmonic acid (JA) signaling is activated in a surrounding ring of cells [1,11,12]. This spatial separation allows SA- and JA-mediated defenses to operate simultaneously during effector-triggered immunity. These insights are reporter-dependent case studies. PR1-driven reporters revealed steep SA halos confined to cells bordering HR lesions, while JA reporters marked a concentric ring beyond, resolving domain-level crosstalk during infection [11,13]. Acinetobacter ADP1 lux biosensors captured early, minute-scale apoplastic SA pulses during resistance responses in tobacco, preceding visible symptoms [2]. Recently, autoluminescent lines enabled continuous whole-plant imaging and multi-day tracking of SA-responsive waves, with tens-of-fold induction ranges upon infection in *Arabidopsis*/*Nicotiana* [3,5,14].

Given SA’s central role in immunity, synthetic analogs have been developed to activate this pathway in crops. Decades of work on SA pathway activators (e.g., the commercial inducer BTH) attest to the importance of SA signaling—these compounds mimic SA to prime broad-spectrum disease resistance and are now used in crop protection [15]. Fundamentally, SA is indispensable for mounting effective immune responses: exogenous SA or SA analogs induce pathogenesis-related (PR) genes, and plants deficient in SA biosynthesis or perception cannot establish robust local or systemic resistance [16]. Accordingly, the key immune regulator NPR1 is required to transduce SA signals and activate downstream defense genes [6].

Interestingly, the efficacy of SA-induced immunity can depend on the time of day, suggesting coordination between defense signaling and the plant’s internal clock. Recent studies indeed demonstrate a tight circadian control of SA responses: SA signaling can alter circadian rhythms via NPR1, and conversely the clock (notably the CCA1 gene) gates SA-induced defense gene expression and disease resistance, peaking at the biologically optimal time [7].

However, applying SA directly can sometimes impair growth, pointing to the need for balanced delivery and hormonal crosstalk management. Notably, encapsulated SA formulations have been shown to bolster plant stress tolerance more effectively than free SA by avoiding excessive SA accumulation and auxin disruption—encapsulated-SA-treated plants maintained normal auxin reporter (DR5) activity under stress, unlike those given free SA [8]. At the molecular level, SA triggers a broad transcriptional cascade that amplifies defense responses. Many transcription factors are induced by SA, enhancing immune signaling. For example, the mulberry gene MiWRKY53—a WRKY transcription factor—is highly SA-inducible (showing ~6–7-fold higher expression with SA treatment) and plays a positive role in defense, as its overexpression elevates PR1 levels and confers enhanced resistance to bacterial infection [9].

Traditional methods like HPLC can measure SA concentrations from harvested tissue, but such chemical assays are destructive and lack spatial information. This limitation has driven the development of non-destructive SA reporters—tools that translate SA presence or signaling into visible signals in living plants [17]. Because SA also orchestrates tolerance to abiotic stresses, SA reporters will be valuable for studying stress hormone crosstalk and resilience in crops [18]. For example, SA has been identified as a key mediator of salt stress tolerance, helping plants maintain ionic balance and activate antioxidant defenses under high salinity [19]. Spatially resolved reporters are especially needed, as hormone pathways often act in distinct domains within tissues. In Arabidopsis, PR1 (SA-responsive) reporters reveal an SA-rich “halo” around infection sites, while VSP1 (JA-responsive) marks a concentric JA zone—a pattern that confirms how these pathways compartmentalize during immune responses [13].

Global transcript profiling further underscores the differences between SA and JA signaling. In soybean, for instance, transcriptome analysis identified over 3100 genes rapidly upregulated by SA (versus ~1450 by JA); SA promptly activated SAR-related genes (e.g., NIMIN1, WRKY40), whereas JA induced later waves of wound-response genes [20]. These distinct gene signatures provide molecular tools for dissecting stress responses and highlight the value of reporters to monitor each pathway. In recent years, interest in visualizing SA in vivo has grown, as live imaging of hormone dynamics can reveal interactions (such as SA–JA crosstalk) and uncover novel defense mechanisms. Beyond its defense roles, SA also profoundly affects plant growth and morphology—high endogenous SA often leads to stunted growth, whereas SA-deficient plants tend to grow larger, and optimal low levels of SA can even promote growth under certain conditions [21]. SA’s interactions with other hormones can be complex: for example, SA and ethylene coordinately promote leaf senescence, with SA-induced chlorophyll loss enhanced by ethylene and requiring the ethylene-regulated transcription factor EIN3 [22].

New reporter technologies with improved sensitivity and resolution have recently become available, expanding the toolkit for plant biologists [23]. Mapping where and when SA accumulates in both model and crop plants may inform strategies for breeding or engineering disease-resistant varieties. Indeed, maintaining appropriate SA levels is crucial to avoid fitness costs: plants with chronically high SA can suffer dwarfism or premature senescence, so SA levels must be finely tuned [24]. Emerging studies have identified new molecular regulators of SA accumulation and revealed roles for SA in processes like leaf aging and nutrient signaling, underscoring that SA’s impact goes far beyond immunity [25]. For example, SA can even modulate secondary metabolism—recent work in Cannabis sativa showed that SA treatment dramatically (≈100–fold) increases expression of key cannabinoid biosynthetic genes (CsPT1 and CsPT4) via specific SA-responsive cis-elements in their promoters [26].

To ensure comprehensive coverage of SA reporter systems in plants, we performed an extensive literature search across multiple scientific databases (including Google Scholar, PubMed, and Elsevier’s ScienceDirect). We used a broad set of keywords and their variants related to salicylic acid and reporter technologies. For example, search terms included combinations like “*salicylic acid reporter*”, “*SA sensor*”, “*SA-inducible promoter*”, “*autoluminescent sensor*”, “*self–luminescent reporter*”, “*SA biosensor*”, “*fluorescent SA sensor*”, “*SA luciferase reporter*”, etc. The search covered publications from early foundational reports to recent studies (up to 2025), and initially yielded hundreds of results.

Our inclusion criteria focused on studies involving plant systems: articles dealing exclusively with non-plant organisms or purely in vitro assays were excluded. Both genetically encoded luminescent reporters (including autoluminescent constructs) and fluorescent reporter systems were considered if used to detect or visualize SA in plants. We also noted related in vivo SA sensing approaches applied in planta (e.g., microbial biosensors or chemical probes) for context. After filtering titles and abstracts, the remaining papers were examined in full to extract information on reporter designs, performance, and applications. This process yielded a curated set of key publications on SA reporters in plants, ensuring that the review reflects the current state of available tools (while acknowledging that some relevant studies might be missed if not indexed in the selected databases or if they employed uncommon terminology).

**Figure 1:**
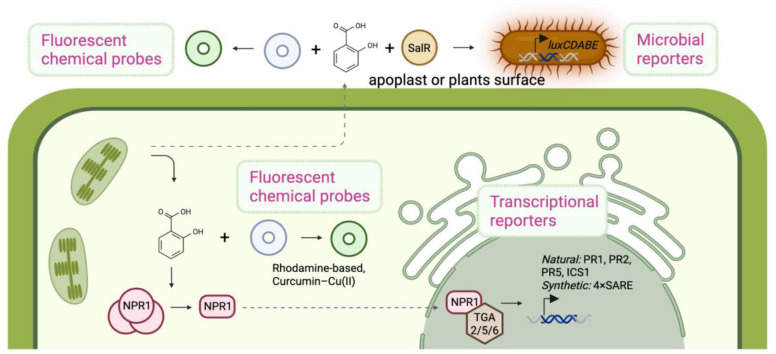
Mechanisms of detection of SA by methods reviewed in this paper.

Multiple types of SA reporter systems have been developed in plant research. These include (Table 1, Figure 1):

1. Transcriptional reporters: Transgenic plants with a native or synthetic SA-inducible promoter driving a reporter gene (such as GUS, GFP, or luciferase). Autoluminescent reporter lines constitute a special subset: the luciferase enzyme and its substrate–biosynthesis pathway are integrated under SA-inducible control, causing the plant to emit light when SA signaling is active [1,3,4,5,10].
2. Microbial biosensors: Engineered bacteria that produce a detectable signal in the presence of SA [2,11].
3. Fluorescent chemical probes: Small molecules that fluoresce or change color upon binding to SA [27].
4. Emerging genetic sensors: New concepts like protein-based or FRET sensors for SA. Genetically encoded SA biosensors remain unrealized in plants largely due to the lack of validated SA-binding scaffolds with suitable affinity/specificity and targeting issues. Developing these sensors is urgent because they would permit single-cell, ratiometric readouts, multiplexing with other hormone reporters, and seamless integration with CRISPR pipelines for high-throughput perturbation screens and precision breeding.

Each type has unique advantages and limitations. Table 1 provides a brief overview of the main classes of SA reporters in plants, highlighting what each measures along with key pros and cons.

To navigate this array of SA reporter systems effectively, this review examines the major classes of plant SA reporters in detail and compares their underlying detection mechanisms and overall performance. It further outlines practical recommendations for selecting appropriate reporter tools and applying them in plant biotechnology and in efforts to enhance stress resilience in crops.

Typical performance ranges (from representative reports): Chemical probes—seconds-to-minutes response; limit of detection (LOD) commonly in the nM range; local, high-resolution readout [11,12,13,14]. Microbial lux biosensors—minutes to respond; nM-level sensitivity for free SA; suitable for rapid quantification on intact tissues or extracts [2,10]. Transcriptional reporters—hours to reach detectable expression; high signal amplification and in vivo context, but indirect and limited for fast transients [1,5,7,8].

## 2. Transcriptional Reporters of Salicylic Acid

Transcriptional reporters are genetically encoded sensors that indicate SA signaling by leveraging the plant’s own SA-responsive gene promoters. In these reporters, an *SA-inducible promoter* (either a native plant promoter from a known SA-responsive gene, or a synthetic promoter engineered to respond to SA) is fused to a reporter gene encoding a visible enzyme or protein. Common reporters include GUS (β-glucuronidase, yielding a blue stain when substrate is applied), GFP/YFP (green/yellow fluorescent protein), or luciferases (e.g., firefly luciferase, producing luminescence in the presence of luciferin). When SA levels rise in the plant, the chosen promoter is activated through the plant’s signaling network, leading to transcription of the reporter gene and a measurable signal in those cells [1].

### 2.1. Mechanism of SA Detection by Transcriptional Reporters

These reporters do not bind SA directly; instead, they monitor the plant’s transcriptional response to SA. At the molecular level, SA accumulation triggers a signaling cascade inside the plant cell. A key player is the NPR1 protein, which acts as a master regulator of SA-mediated gene expression. In the presence of SA, NPR1 undergoes a conformational change and moves to the nucleus, where it interacts with TGA transcription factors to activate pathogenesis-related (PR) gene promoters [13]. Promoters of SA-responsive genes (such as PR1, PR2, PR5, or ICS1) contain specific DNA motifs (e.g., the TGACG motif, also called an “as-1” or TGA-element) that are recognized by these transcription factors. NPR1 serves as a co-activator, recruiting the transcriptional machinery to induce these genes [13]. In a transgenic reporter line, the same promoter (for example, the PR1 promoter) is placed upstream of a reporter gene. Thus, when SA causes NPR1 and other factors to activate the PR1 promoter, the reporter gene is expressed instead of the native PR1 gene, producing a detectable signal. This chain of events means the reporter’s output is an indirect proxy for SA—it reports that “SA signaling is active” (i.e., downstream defense genes are being turned on), rather than measuring SA molecules themselves [1,13].

Why transcriptional reporters are temporally slower. Transcriptional systems are inherently multistep: SA accumulation must (1) activate NPR1 and associated TFs, (2) trigger promoter occupancy, (3) drive transcription and mRNA processing, (4) translate reporter protein, and (5) (for some readouts) allow reporter maturation or substrate application. This cascade typically yields detectable signals on the order of hours, whereas microbial biosensors respond within minutes (direct SA sensing by a bacterial regulator driving lux output) and fluorescent chemical probes can switch within seconds due to immediate binding/chemical reaction with SA [2,10,11,12,13,14]. Functionally, transcriptional reporters measure pathway activation, while microbial/probe systems measure SA itself, explaining the gap in temporal resolution.

### 2.2. Natural Promoter Reporters of SA

The simplest and most widely used transcriptional reporters use native SA-responsive promoters from plants. For instance, the promoter of the *PR1* gene (a classic SA-inducible defense gene in Arabidopsis) is frequently used. In a *PR1::GUS* line, blue staining indicates where the PR1 promoter is active, which in turn reflects where SA signaling has occurred. These natural promoter fusions have several strengths. Because they are actual promoters from SA-responsive genes, they provide a biologically contextual readout of the plant’s immune response. The reporter expression will occur in the same cells where an SA-responsive defense gene would normally turn on, giving a spatially accurate picture of hormone signaling. Indeed, promoter reporter lines have shown high spatial resolution—from specific cell layers up to whole-organ patterns—of SA-driven gene activation. Another benefit is signal amplification: a strong promoter like *PR1* can drive high levels of the reporter enzyme/protein when induced, resulting in a robust signal (for example, bright fluorescence or luminescence). Transcriptional reporters are well-established in model plants such as *Arabidopsis thaliana* and *Nicotiana benthamiana*, and have been used to visualize spatiotemporal patterns of SA signaling during pathogen infection. For example, transgenic Arabidopsis carrying a *PR1::YFP* reporter shows defined halos of fluorescence around infection sites, corresponding to the zone of elevated SA signaling in those tissues. Likewise, *PR1::LUC* (luciferase) lines have been used to track the spread of SA-induced gene expression over time: when a leaf is infected, over the next 1–2 days, luciferase activity spreads to neighboring leaves as the SA signal (and SAR induction) propagates. These kinds of experiments demonstrate how natural promoter reporters can map the dynamics of SA-mediated defense activation in vivo [1].

Despite their usefulness, natural promoter reporters have important limitations. First, they measure SA signaling indirectly. There is an inherent time lag: SA must accumulate and trigger the signaling network, transcription factors must activate the promoter, the reporter mRNA and protein must be produced, and (for some reporters) a substrate must be applied before a signal is seen. This process typically takes hours, so transcriptional reporters cannot capture sudden changes in SA levels in real time. Additionally, because they rely on a plant gene promoter, the readout can be influenced by crosstalk with other signals. Many SA-inducible promoters also respond to other defense hormones or stress conditions. For instance, the *PR1* promoter, while largely SA-responsive, can be modestly induced by certain forms of stress or by high levels of related signals like jasmonates or ET, especially in mutants or particular contexts. This means a reporter signal might not strictly indicate “SA present” but rather “SA pathway active,” which could sometimes be triggered by SA-independent pathways. Careful controls are needed to interpret results (e.g., confirming that the reporter is not induced in mutants that cannot accumulate SA). Another drawback is the need to generate transgenic plants. Introducing a promoter reporter construct into the plant genome requires transformation and breeding, which can be laborious, especially in crop species. Moreover, some reporter assays are invasive or end-point: for example, GUS staining requires sacrificing the tissue and cannot be observed in the same sample over time, while luciferase imaging needs addition of the luciferin substrate to the plant, which can be cumbersome for long-term or high-throughput experiments [1].

*Autoluminescent* SA reporters are an innovative variant of the transcriptional reporter approach that address some of the above limitations. In these systems, instead of a single reporter enzyme, an entire bioluminescence pathway is engineered into the plant under control of an SA-responsive promoter. Recent examples use a fungal luciferin biosynthesis pathway (from *Neonothopanus* mushrooms) integrated into plants. For instance, researchers have placed a fungal luciferase enzyme gene and the genes for its substrate synthesis under the *WRKY70* promoter in Arabidopsis and tobacco [4]. When SA signaling activates the promoter, the plant begins producing the luciferase, while luciferin is produced constitutively. This causes the plant to emit visible light without any external substrate. This is essentially a self-contained luciferase reporter. The major advantage of autoluminescent lines is that they enable continuous, non-invasive monitoring of SA-responsive gene activity in real time. The plants literally glow when and where SA signaling is active, allowing researchers to track hormone waves or oscillations over long periods (days to weeks) with minimal disturbance. The background is essentially zero (plants do not naturally luminesce), and induction can produce tens-of-fold increases in light output; in one demonstration, SA-inducible autoluminescent plants showed up to ~50-fold higher luminescence after infection, easily captured by standard cameras. Entire seedlings or canopies have been imaged to watch SA signaling spread during immune responses. Autoluminescent reporters thus combine the specificity of a transcriptional reporter with the convenience of a substrate-free luminescence readout, making them a powerful new tool.

Recent advances in luminescence-based salicylic acid imaging include the development of a ratiometric dual-color reporter based on a fungal bioluminescence system [5]. One color reports the activity of the SA-responsive promoter, while the second is driven by a constitutive reference promoter. Because metabolic fluctuations affect both channels similarly, the intensity ratio provides a normalized, quantitative ratiometric readout. This makes the technology the first non-invasive quantitative luminescence-based approach enabling tissue and whole-plant imaging of salicylic activity in planta.

The drawbacks, however, include the complex engineering required (introducing multiple genes and pathways into the plant) and the fact that the luminescence, while visible, is relatively dim compared to traditional luciferase assays with an added substrate. Sensitive cameras and dark imaging conditions are usually required to detect the glow. Additionally, like other promoter fusion reporters, autoluminescent systems still report on SA signaling rather than measuring SA directly. Nonetheless, as the technology matures, these reporters are poised to become valuable for whole-plant, long-term imaging of defense hormone dynamics.

### 2.3. Synthetic Promoter Reporters of SA

Another branch of transcriptional reporters uses synthetic promoters that are artificially designed to respond to SA. These constructs typically multimerize known SA-responsive cis-elements (DNA motifs) and combine them with a minimal promoter to drive a reporter gene. For example, researchers have created synthetic promoters containing repeated copies of the SARE (salicylic acid responsive element) sequence or the as-1 element, which are DNA motifs recognized by the SA pathway transcription factors (such as TGA factors). By placing, for example, four tandem SARE copies upstream of a minimal promoter and a luciferase gene (4×*SARE::LUC*), one can create a module that is highly sensitive to SA signaling. The mechanism is fundamentally the same as with natural promoters—the synthetic promoter is activated by NPR1-dependent transcription factors in the presence of SA—but the response can be quantitatively different. Advantages of synthetic promoters include enhanced sensitivity and customizable specificity [15]. With multiple high-affinity binding sites for SA-activated transcription factors, the synthetic promoter can amplify the transcriptional response, leading to stronger reporter expression than a single native promoter might produce. This can be very useful if the native promoter is weak or if one needs to detect faint signals. Synthetic constructs can also be engineered to reduce cross-reactivity; for instance, one can omit undesired regulatory elements or combine elements to respond predominantly to SA and not to other stimuli. Moreover, synthetic promoters are modular—they can be transferred into different plant species or tuned by mutating binding sites. There are examples of hybrid promoters built to function as SA sensors in crops like potato, achieved by adjusting the sequence context so that the plant’s transcription factors recognize them optimally.

On the downside, synthetic promoter reporters share some limitations with natural promoter fusions and introduce a few of their own. They are still an indirect measure of SA: like any transcriptional reporter, they report the activation of a signaling pathway, not the actual concentration of SA. If SA signaling is blocked or altered, the synthetic promoter will not light up even if SA is present (and conversely could potentially be triggered by pathway crosstalk if not perfectly specific). Additionally, a synthetic promoter may not perfectly reproduce the nuanced expression pattern of a native promoter. Real gene promoters often have complex regulation (chromatin context, multiple enhancers, tissue-specific repressors, etc.) that a simplified synthetic construct might not capture. Thus, a synthetic reporter might show SA responsiveness, but perhaps not only in the exact cells or conditions that a native promoter would—it could be “leaky” or miss some spatial control. Each new promoter design also requires validation in planta: one must test that it truly responds to SA and not to other signals in the target plant, which can be time-consuming. Finally, creating an optimized synthetic promoter often involves trial-and-error and iteration of designs. Despite these caveats, synthetic promoters are extremely useful when native options are lacking or when one needs extra sensitivity. They provide a way to boost signal output or adapt SA reporters to non-model species where the standard Arabidopsis PR1 promoter might not work efficiently.

### 2.4. Strengths of Transcriptional Reporters

A major strength is biological context: a PR1 or similar SA-inducible promoter will activate the reporter in the same cells where endogenous defense genes turn on, yielding a highly spatially resolved map of SA signaling. Strong promoters can amplify the signal, producing bright fluorescence or luminescence upon induction. Synthetic promoter fusions (for example, multimerized SA-responsive cis-elements) can further boost sensitivity and reduce background, enabling detection of very low SA levels. Autoluminescent variants integrate a full bioluminescent pathway under SA control, so the plant emits light without added substrate: these plants literally “glow” in SA-rich tissues with minimal background and large fold-changes upon defense activation. In sum, transcriptional reporters provide in vivo, high-resolution, non-destructive readouts of SA activity with strong signal amplification, making them well suited for mapping hormone dynamics in model plants.

### 2.5. Limitations of Transcriptional Reporters

Despite their description in Section 2.4., transcriptional reporters have intrinsic limitations. First, they report SA signaling rather than SA itself, so there is a significant time lag: hormone accumulation must activate NPR1 and transcription factors, drive transcription and translation, and (for luciferases with undeciphered luciferin biosynthesis pathway) require substrate application. This multistep process typically takes hours to generate a detectable signal, precluding real-time monitoring of rapid SA fluctuations. Second, promoter crosstalk can reduce specificity: many “SA-inducible” promoters are also weakly responsive to other stresses or hormones (e.g., jasmonate, ethylene), so a reporter may indicate “pathogen response on” even if SA levels are unchanged. Third, these reporters require transgenic plants and often invasive assays. Generating stable lines is laborious in non-model species, and some readouts are destructive or endpoint (for example, GUS staining kills tissue; conventional luciferase imaging requires exogenous luciferin). Even autoluminescent systems, while substrate-free, demand complex multi-gene engineering and very sensitive cameras (luminescence is relatively dim). Thus, although powerful for qualitative and spatially resolved mapping, transcriptional reporters are comparatively slow and indirect, and they do not easily provide absolute SA concentrations.

In summary, transcriptional SA reporters offer biologically contextual, high-spatial-resolution readouts of pathway activation, leveraging native or synthetic SA-responsive promoters to amplify signal and enable whole-plant imaging (including substrate-independent autoluminescent variants and ratiometric designs that improve normalization); however, they are inherently indirect and temporally delayed (requiring NPR1/TGA activation, transcription/translation, and sometimes reporter maturation), susceptible to promoter crosstalk and chromatin/context effects, and often demand transgenesis and species-specific validation, while some formats remain substrate-dependent and generally less suited for absolute quantification or rapid, transient SA fluctuations.

## 3. Microbial Biosensors

Microbial SA biosensors are living reporter systems that detect salicylic acid using engineered bacteria. Unlike plant transcriptional reporters (which rely on the plant’s internal signaling to indirectly report SA), these biosensors directly sense SA molecules and produce a measurable output (commonly light). The general approach is to take a non-pathogenic bacterium and equip it with a genetic circuit that responds to SA. A well-established example is the *Acinetobacter baylyi* ADP1 lux biosensor. In this system, the soil bacterium *A. baylyi ADP1* is genetically modified to carry a luminescence (lux) operon under the control of an SA-responsive promoter. Specifically, *A. baylyi ADP1* has a native regulatory protein (often referred to as SalR or a similar transcription factor) that can bind salicylic acid. When SA is present, the SA-bound regulator activates the luxCDABE operon (the genes that produce bacterial luciferase and its substrate), causing the bacteria to emit light. The intensity of luminescence is proportional to the SA concentration that the bacteria encounter. Importantly, this detection is quantitative and real-time—as SA levels rise or fall in the environment, the bacterial light output changes accordingly, without needing any additional reagents [16].

### 3.1. Mechanism of Microbial Sensors

To use the microbial sensor, researchers typically either spray or gently infiltrate the modified bacteria onto plant tissue (for example, onto a leaf surface or into the leaf apoplast). The bacteria settle on the plant tissue and act as tiny SA detectors. If the plant tissue contains SA (or the volatile methyl salicylate, which many sensors also detect after it diffuses and is converted to SA by the bacteria), the microbes will start glowing. By measuring the light (either with a low-light camera or a luminometer for harvested samples), one can determine the local SA level. Because the luminescence response is rapid (often detectable within minutes) and roughly proportional to SA over a certain range, microbial biosensors can provide a direct readout of SA concentration in situ [16].

### 3.2. Strengths of Microbe-Based Sensors

A major advantage of microbial SA biosensors is that they measure free SA directly, rather than a downstream biological response. This can make them more specific in some contexts—they respond to the presence of the SA molecule itself. They are also highly sensitive. Some bacterial sensors can detect SA on the order of nanomoles or even lower, revealing tiny amounts of the hormone. In practical terms, the microbial luminescence assay can register an increase in SA within a plant tissue often before traditional methods would detect it. For instance, using the *Acinetobacter* lux sensor, researchers demonstrated that tobacco leaves undergoing resistance responses have transient SA flashes in the apoplast even before visible disease symptoms develop. This shows the power of the sensor to catch early or subtle SA production. Another benefit is that these biosensors are non-destructive to the plant. The bacteria can be applied to an intact leaf, and SA levels can be monitored over time without sacrificing the tissue (unlike HPLC, which requires tissue extraction). This allows time-course measurements in vivo. Furthermore, microbial sensors are quite versatile and portable—they can, in principle, be used on any plant species because they do not require plant genetic transformation. This makes them handy for high-throughput screening of SA levels across different genotypes or treatments: for example, one can take leaf disks from various plants, add the sensor bacteria, and use a plate reader to compare SA production across samples [2,11].

### 3.3. Limitations of Microbial Sensors

Despite their utility, microbial SA reporters have notable limitations. One is that the bacteria must physically access the SA. Detection is limited to where the microbes can reach, typically the surface of a leaf or the spaces between cells (apoplast) if infiltrated. They cannot easily go deep into intact tissues or inside cells. Thus, they might miss SA that is present in internal organs or tightly sealed compartments. If one needs to measure SA within the phloem or inside roots without exposing them, microbial sensors are not suitable. Another limitation is temporal: while the sensor can report changes over time, it is not ideal for very long-term experiments on the same plant because the plant’s immune system may eventually recognize and respond to the introduced bacteria. Over hours or days, the plant might ramp up defenses that inhibit or kill the sensor microbes, or the bacteria might simply not survive well, leading to signal loss. For example, an initial luminescence might wane after a day as the bacteria are cleared or nutrients are exhausted. Therefore, microbial sensors are great for short-term monitoring (minutes to hours, or perhaps a day) but not for continuous multi-day imaging of the same sample. Another practical drawback is that using these sensors involves handling live genetically modified organisms and requires specialized low-light imaging equipment. Working with a luminescent bacterium means one must take biosafety precautions and have a sensitive camera or luminometer. This can complicate experiments compared to purely genetic plant reporters or chemical probes. Finally, while generally specific, some bacterial sensors might respond to salicylates broadly (e.g., they might also detect very closely related chemicals or require metabolic conversion of SA conjugates), so one must ensure the assay conditions (like enzymatically cleaving SA glucose conjugates if the goal is total SA measurement) are appropriate [2].

In summary, microbial SA biosensors offer a direct and sensitive way to quantify SA in planta in real time. They complement plant-based reporters by providing quantitative data on actual SA levels and are especially useful for rapid assays or for plants where creating transgenics is not feasible. However, they are best suited for accessible tissues and relatively short observations, and they operate outside the plant’s cells, which means they report extracellular SA (or SA that diffuses out of cells) rather than internal signaling status [2,11].

## 4. Fluorescent Chemical Probes

Fluorescent chemical probes for salicylic acid are small molecules that undergo a detectable change (usually in fluorescence or color) upon binding or reacting with SA. These are essentially synthetic chemical sensors: researchers design a molecule that can specifically recognize SA (for example, by having a binding pocket or a chemical reaction with SA) and then transduce that recognition into a fluorescence “turn-on” or color shift. Such probes enable visualization or quantification of SA without genetic modification of the plant.

### 4.1. Mechanism of Detection

A typical fluorescent SA probe is a compound that is initially non-fluorescent or weakly fluorescent but becomes highly fluorescent in the presence of SA. One strategy is to use a fluorescence quencher that is released when SA interacts with the probe. For instance, a rhodamine-based SA probe was developed that remains colorless and non-fluorescent until it encounters SA; the SA triggers a structural change (like opening a spiro-lactone ring in the rhodamine) that turns the fluorescence “on” [6]). Another example is a curcumin–Cu(II) ensemble probe: curcumin (a naturally fluorescent compound) is complexed with copper ions, which quench its fluorescence; when SA is present, it has a higher affinity for Cu(II), so it pulls the copper away from curcumin, allowing curcumin to fluoresce [7]. In both cases, the result is a fluorescent signal whose intensity is proportional to the amount of SA that the probe has encountered. Some probes also produce a visible color change (e.g., a shift from colorless to pink) upon binding SA, which can be observed by eye or simple instruments [7,8].

To use these probes, researchers typically apply them to plant tissues by infiltration or incubation. For example, leaves can be dipped or pressure-infiltrated with a solution of the probe, or the probe can be fed to whole seedlings via the roots. After allowing time for the probe to diffuse and equilibrate, any SA present in the tissue will react with or bind to the probe, causing the fluorescence to appear in those locations. The plant can then be examined under a fluorescence microscope or imaging system to see where the probe lights up, indicating the presence of SA.

### 4.2. Strengths of Fluorescent Chemical Probes

Fluorescent probes offer several compelling advantages. They are often extremely sensitive and fast. Some SA probes can detect very low concentrations (nanomolar or even lower) of SA and respond within seconds to minutes. This high sensitivity can surpass that of genetic reporters, allowing detection of faint SA signals that might not induce a noticeable gene expression change. Additionally, because they are small molecules, probes can sometimes reach cellular and subcellular locations that are challenging for other reporters. With appropriate preparation, probes can be taken up into tissues, enabling high-resolution mapping of SA at the microscopic level. Researchers have used fluorescent probes to visualize SA accumulation in specific cells and even organelles. For example, a curcumin-based probe was used to stain *Arabidopsis* leaf tissues and roots, revealing distinct patterns of SA localization inside living cells. This kind of fine-scale localization is difficult to achieve with whole-organism reporters. Some probes also have the benefit of producing a direct readout (they respond to SA itself, not via a biological circuit) and can be applied to any plant species or sample. There is no need to create transgenics or crosses; even crop species or field samples can potentially be tested by applying a probe. Moreover, certain probes yield changes visible under simple conditions (e.g., fluorescence under a handheld UV lamp or a color change visible to the naked eye), which makes them accessible for quick assays without sophisticated equipment. They are ideal for confirming the presence of SA “hotspots” in tissue samples and can complement other measurements by validating where SA has accumulated [7].

### 4.3. Limitations of Fluorescent Chemical Probes

The use of chemical probes also comes with important caveats. A key limitation is that the probe must be physically introduced into the plant tissue, which can be somewhat invasive. Infiltrating a leaf with a solvent or buffer containing the probe might itself induce some wounding or stress responses. Also, uptake of the probe may be uneven—some regions of tissue may get more of the probe than others, leading to non-uniform staining. Researchers must ensure that the probe actually reaches the areas of interest; for example, thicker or waxy tissues might be hard to penetrate. Another limitation is that most probe reactions are single-use or irreversible. Once the probe molecule has interacted with SA and changed its fluorescence, it typically cannot be “reset.” This means that if SA levels change over time, a given probe molecule will not indicate a decrease (it will just stay in its activated state or remain bound). Practically, this limits time-course studies: one cannot easily monitor the same sample continuously, because the probe’s first encounter with SA produces a permanent signal. To get a time-series, one would need to use multiple samples or reapply the probe at different time points (which might not be equivalent conditions). Additionally, fluorescence can photobleach or fade over time, especially under microscope illumination, which can complicate quantification in prolonged imaging sessions. Another consideration is specificity: probes are designed to be selective for SA, but they could have potential side reactions or might respond to very similar molecules in some cases. Rigorous testing is needed to ensure they do not give false positives with compounds other than SA. Finally, compared to genetic reporters or microbial sensors, chemical probes are typically less suited for quantitative measurement of SA concentrations in bulk (though there are spectroscopic methods to quantify fluorescence intensity, the readout may be influenced by probe distribution and tissue optics). They are most powerful for visualization and relative comparisons (e.g., comparing fluorescence intensity between treatments as a proxy for more or less SA) [2].

In summary, fluorescent SA probes are valuable tools for detecting SA with high sensitivity and spatial resolution. They provide a direct chemical readout and can reveal where SA accumulates within plant tissues at the micro-scale. Their use is ideal for short-term experiments and imaging of SA distribution in situ. However, because they require external application and generally provide a one-time snapshot of SA, they are often used to complement other methods (for example, to validate and fine-tune the findings from genetic reporters or to do detailed imaging after an initial survey of SA levels by other means). Combining a probe with microscopic analysis has allowed researchers to confirm, for instance, that SA concentrates in certain cells or organelles during immune responses—information that enhances our understanding of hormone physiology beyond what whole-plant reporters can show.

## 5. Emerging Genetic Sensors

Given the success of reporters for other hormones (such as auxin, ABA, calcium, etc.), there is interest in developing genetically encoded sensors that can directly sense SA and report it, for example, via a fluorescence resonance energy transfer (FRET) signal or another real-time output. An ideal SA biosensor of this kind would be a fusion protein that changes its fluorescence when it binds SA, allowing live measurement of SA concentrations in real time inside plant cells. Such a tool could, in principle, be targeted to specific subcellular compartments (nucleus, chloroplast, etc.), be reversible, and give a quantitative ratiometric readout [11].

### 5.1. Concept and Potential

Imagine a protein that has two fluorophores connected by an SA-binding domain—binding of SA brings the fluorophores closer and produces a FRET signal. If achieved, this would provide a direct and real-time readout of SA levels in vivo. Unlike transcriptional reporters, it would not be limited by gene expression or by indirect signals; it would report the actual concentration of SA (like a molecular thermometer for SA). This could reveal rapid SA fluctuations and allow continuous monitoring with subcellular precision. Researchers have successfully made such sensors for other small molecules (e.g., the ABA biosensor ABAleon, or calcium sensors like Cameleons), so it is a tantalizing prospect to have one for salicylic acid [11].

### 5.2. Challenges and Status

To date, no fully functional genetically encoded SA sensor has been reported. SA has proven to be a difficult target for a few reasons. First, the known SA-binding proteins in plants, such as NPR1 (and its paralogs NPR3/NPR4) or certain SABPs (salicylic acid-binding proteins), have not yet been successfully engineered into sensors. NPR1 itself is a cofactor protein that does not undergo a simple obvious conformational change upon SA binding that could be easily linked to a fluorescent output (its activation involves oligomer–to–monomer conversion and interaction with DNA-binding factors, a complex process). Attempts to use NPR1 in biosensor designs have not yielded a clear in vivo SA sensor; NPR1 is also quite large and has multiple roles, which complicates using it as a simple ligand-binding domain. Other candidates, like certain enzymes that bind SA, either bind too weakly or are not specific enough. As a result, current efforts are still largely experimental. Some researchers have used creative indirect genetic approaches as a stopgap. For example, one workaround has been to monitor the SA-triggered nuclear translocation of an NPR1–GFP fusion protein. In this method, you create transgenic plants where NPR1 is tagged with GFP, and you image cells to see when GFP moves from the cytoplasm to the nucleus (which happens when SA levels rise and NPR1 is activated). This can indicate an SA increase, but it is obviously indirect and not quantitatively precise—it is essentially a binary or qualitative indicator and still relies on the native signaling pathway. Another example is using promoter reporter fusions of multiple SA marker genes and computationally integrating them, but that again is indirect [1,11,13].

The absence of a true genetically encoded SA sensor highlights a gap in the current toolkit. It is an active area of research, and any breakthrough (for instance, a successful FRET sensor protein that binds SA specifically) would be rapidly adopted. Until then, plant biologists rely on the combination of transcriptional reporters, chemical probes, and other methods discussed above to infer SA dynamics. The hope is that continued research into SA’s molecular targets (for example, newly discovered binding proteins or engineered variants of NPR1 or other receptors) could eventually yield a sensor. Such a sensor could theoretically be introduced into plants via transformation or even transient expression, enabling real-time tracking of SA at the cellular level, similar to how we can track calcium or ATP changes in living cells.

## 6. Discussion and Recommendations

No single SA reporter is perfect for all purposes—each has trade-offs in sensitivity, specificity, invasiveness, and practicality. The optimal choice depends on the experimental question and plant system. Classical promoter reporter lines (for example, Arabidopsis plants carrying a PR1::GUS or PR1::LUC transgene) remain popular for visualizing where SA-responsive genes are activated in planta. These reporters reflect authentic downstream responses to SA and have been indispensable for mapping spatial patterns of SA signaling over time [28]. However, because they indicate SA signaling (gene expression) rather than free SA, their readout can be influenced by other factors—for instance, other defense pathways that also induce PR gene expression. In practice, researchers often use promoter fusion lines to observe how SA responses spread during infections; a PR1::LUC transgenic will luminesce in the infected leaf and neighboring tissues, revealing the progression of SA-mediated defense from an initial infection site into surrounding areas over days. It is worth noting that very high levels of SA can directly interfere with some reporters—SA treatment is known to quench GFP fluorescence in vivo, so fluorescence-based reporters must be interpreted with caution or paired with SA-insensitive variants like RFP.

By contrast, chemical probes and microbial biosensors offer more direct measurements of SA itself. A well-known example is the *Acinetobacter* ADP1 lux biosensor, which has been used to quantitatively map SA accumulation in tobacco leaves after pathogen attack, revealing transient SA pulses in the leaf apoplast even before visible symptoms and capturing early dynamics of hormone release [2]. Similarly, synthetic fluorogenic probes can pinpoint SA “hotspots” with high sensitivity and spatial resolution. For example, a curcumin-based fluorescent probe visualized SA inside living *Arabidopsis* leaf tissues and roots [7]. Such tools are excellent for short-term, ultra-sensitive detection of SA (down to picomole levels) [9] and for confirming SA accumulation in specific cells or compartments. Their invasive application and one-time-use nature make them less suitable for continuous monitoring, but they are ideal for snapshot experiments or for validating SA distribution at particular moments.

The newest generation of SA reporters is the autoluminescent transgenic plant lines, which are especially powerful for long-term and whole-plant monitoring. By integrating a fungal bioluminescence pathway under an SA-inducible promoter, researchers have created plants that literally glow when SA signaling is active [4]. In recent demonstrations, SA-inducible autoluminescent Arabidopsis and tobacco lines showed over 50-fold increases in luminescence upon pathogen infection, allowing entire plants to be imaged in real time with standard cameras [5]. This non-invasive approach enables tracking of SA signaling waves (for example, during development of SAR or even across day–night cycles) over days to weeks. The complexity of generating these lines (multi-gene engineering) is a consideration, but once established, they provide a unique window into hormone dynamics at the whole-plant scale.

We believe that autoluminescent lines outperform GUS for longitudinal imaging. GUS histochemistry is endpoint and invasive (tissue sacrifice, substrate incubation), precluding time-series in the same organ; by contrast, autoluminescent systems are substrate-independent and non-destructive, enabling continuous imaging over days or weeks with near-zero background. In infection assays, SA-inducible autoluminescent plants showed >10- to ~50-fold increases in photon output, supporting canopy-scale visualization of signal propagation and circadian gating without perturbation [3,5,10]. Ratiometric dual-color designs further normalize metabolic drift, improving quantitative comparability across tissues and time points [5]. Together, these features explain superior performance in long-term, whole-plant studies compared with GUS.

Given the diverse toolkit available, investigators should choose the SA reporter system best suited to their needs—and often multiple approaches can be combined to gain complementary insights. For instance, one might use an autoluminescent reporter line to monitor overall SA responses in a plant, and simultaneously apply a fluorescent probe or a microbial sensor to quantify actual SA levels in specific tissues.

In summary, each reporter type has its niche, and using them appropriately can greatly enhance our understanding of SA biology. Some practical guidelines are summarized in Table 2.

## 7. Conclusions

As of 2025, autonomous luminescent transgenic plants have emerged as arguably the most powerful and versatile SA reporters for live imaging. These autoluminescent, ratiometric systems overcome many limitations of earlier reporters—requiring no external substrates and causing minimal perturbation to the plant—making them a new gold standard for observing hormone activity in vivo [5]. Incorporating a full luciferin biosynthesis pathway (fungal bioluminescence, FBP) under an SA-inducible promoter eliminates the need for exogenous luciferin and allows flexible promoter swapping to program hormone-dependent glow patterns across organs, enabling low-cost, long-duration imaging of SA signaling [10]. For example, Khakhar et al. (2020) demonstrated a synthetic SA-responsive luciferase system that renders plants self-luminous during immune responses [10].

At the same time, no single reporter is “best” in all contexts. The optimal choice depends on the experimental goal. For instance, a simple bacterial biosensor or a fluorescent probe might be ideal for a quick measurement of SA in a leaf extract, whereas luminescent reporter lines excel at monitoring dynamic defense responses inside an intact plant. In practice, using multiple complementary reporters in parallel can provide the most complete picture of SA biology [27]. By combining approaches—for example, employing an autoluminescent line to track whole-plant SA signaling and a microscopy-compatible fluorescent probe to visualize cellular-level SA accumulation—researchers can cross-validate results and overcome the limitations of any single method [29].

Looking ahead, a truly direct, genetically encoded SA biosensor (for example, a FRET-based protein that changes its fluorescence upon binding SA) remains a major goal for the field. Recent reviews emphasize both the promise and the current gaps: notably, plant biosensor technologies still lack a direct SA sensor, even as advances like autoluminescent reporters and multi-gene expression strategies (e.g., 2A peptide-linked reporters) are moving the field forward [18,19]. Success in developing such a sensor would enable precise real-time quantification of SA in specific cells and subcellular compartments, further enhancing our ability to monitor this crucial hormone. Ongoing progress in synthetic biology and protein engineering may eventually yield a true genetically encoded SA sensor [30]. In the meantime, plant scientists now have an expanding and impressive toolkit of SA reporters at their disposal. Thoughtfully choosing and combining these tools will continue to illuminate how salicylic acid orchestrates plant defense, and will aid efforts to translate that knowledge into improved crop resilience [1,31,32,33,34,35]. Beyond research applications, SA also underpins aspects of post-harvest physiology: exogenous SA treatments can improve product quality and disease resistance, highlighting the translational value of SA monitoring for crop storage and protection [20,21,36].

## Figures and Tables

**Table 1:** Summary of salicylic acid (SA) reporter systems in plants.

Reporter Type	Concentration of SA Detected	Speed of SA Detection	Specific to SA?	Requires Transgenic Lines?	Depends on the Plant Species?	Long-Term Imaging	Non-Invasive?	Quantitative?	Parts of Plant Visualized	Other Features	Citation
Transcriptional reporters (natural promoter fusions) (e.g., *PR1::GUS, ICS1::LUC*)	**Depends** on a promoter used	**Slow**—typically 6–24 h post-challenge	**Not fully**—promoter crosstalk	**Yes**	**Yes**	**Yes**	Generally—**no**, autoluminescent reporters—**yes**	Generally—**no**, autoluminescent ratiometric reporters—**yes**	Throughout the plant—high spatial resolution	–Strong signal amplification; –In vivo contextual readout	[1]
Transcriptional reporters (synthetic promoters) (e.g., 4×*SARE::LUC*)	**Depends** on a promoter used	**Faster than natural promoters**—1–6 h post-challenge	**Yes**—reduced crosstalk	**Yes**	**Adaptable**—but requires validation	**Yes**	Generally—**no**, autoluminescent—**yes**	Generally—**no**, autoluminescent ratiometric reporters—**yes**	Throughout the plant—high spatial resolution	–Enhanced sensitivity; –May not mimic native expression patterns	[15]
Microbial biosensors (e.g., *Acinetobacter ADP1* + salicylate-inducible *lux*)	**Sub-µM** in vitro; higher in planta (context-dependent)	**Fast**—within minutes	**Yes**	**No**	**No**	**No**—host clears the bacteria	**No**	**Yes**	**Local**—limited to surface/apoplast SA: bacteria must contact SA	–Requires handling GM microbes and low-light imaging equipment	[16]
Fluorescent chemical probes (e.g., rhodamine-based turn-on dye, curcumin–Cu(II) ensemble)	**nM–µM;** often **µM** effective in tissues	**Fast**—within seconds	**Yes**	**No**	**No**	**No**—one–time use	**No**	**No**	**Local**—high-resolution visualization of SA distribution (microscopic imaging)	–Some probes give visible readouts (e.g., fluorescence under UV)	[6,7]

**Table 2:** Suggested SA reporters for various experimental scenarios.

Scenario	Recommended Reporter(s)	Why
Analyzing spatial patterns of SA signaling (e.g., which cells activate defenses during infection)	Transcriptional promoter reporter lines (e.g., *PR1::LUC*, plus *PR1::GUS* for endpoint staining)	Visualizes where SA-responsive genes are activated in planta, with tissue-level detail. Non-destructive imaging over time (luciferase) plus high-resolution end-point confirmation (*GUS* histochemistry).
Continuous, multi-day monitoring of SA dynamics (e.g., tracking a SAR signal wave)	Autoluminescent ratiometric SA reporter lines	Allows whole-plant, real-time imaging of SA signaling waves without disturbing the plant (no need for external substrates or sampling). Ideal for observing hormone fluctuations and systemic responses over time.
Rapid or high-throughput SA quantification (many samples, or non-model species)	Microbial *lux* biosensor assays (e.g., *Acinetobacter* ADP1 applied to leaves or extracts)	Provides a direct, sensitive readout of SA levels. Can be applied to diverse plants without genetic modification. Suitable for comparing SA amounts across treatments or genotypes using plate readers or imaging systems.
Subcellular localization/fine-scale mapping of SA	Fluorescent chemical probe with microscopy	Offers microscopic resolution to pinpoint SA within cells or tissues. Useful for confirming SA accumulation in specific sites (e.g., chloroplasts, phloem) via fluorescence imaging.
Adapting SA reporters to a new plant species (or boosting reporter sensitivity)	Synthetic SA-responsive promoter constructs	Modular designs can be tuned and introduced into the species of interest. Can increase signal strength or specificity where native promoters are weak or unresponsive. Helpful in non-model plants if standard promoters do not work optimally.

## Data Availability

The original contributions presented in this study are included in the article. Further inquiries can be directed to the corresponding author.

## Abbreviations

The following abbreviations are used in this manuscript:

ABA	abscisic acid
ABAleon	abscisic acid FRET biosensor ABAleon
ADP1	*Acinetobacter baylyi* ADP1
as–1	activation sequence-1 element
ATP	adenosine triphosphate
DNA	deoxyribonucleic acid
ET	ethylene
FRET	fluorescence resonance energy transfer
GFP	green fluorescent protein
GM	genetically modified
GUS	β-glucuronidase
HPLC	high-performance liquid chromatography
ICS1	ISOCHORISMATE SYNTHASE 1
LUC	luciferase
luxCDABE	bacterial luciferase operon luxCDABE
NPR1	NONEXPRESSOR OF PATHOGENESIS-RELATED GENES 1
NPR3	NONEXPRESSOR OF PATHOGENESIS-RELATED GENES 3
NPR4	NONEXPRESSOR OF PATHOGENESIS-RELATED GENES 4
PR	pathogenesis-related
PR1	PATHOGENESIS-RELATED 1
PR2	PATHOGENESIS-RELATED 2
PR5	PATHOGENESIS-RELATED 5
RFP	red fluorescent protein
SA	salicylic acid
SAR	systemic acquired resistance
SARE	salicylic acid responsive element
TGA	TGA transcription factor
UV	ultraviolet
WRKY70	WRKY transcription factor 70
YFP	yellow fluorescent protein
